# The influence of a high intensity physical activity intervention on a selection of health related outcomes: an ecological approach

**DOI:** 10.1186/1471-2458-10-8

**Published:** 2010-01-08

**Authors:** Duncan S Buchan, Stewart Ollis, Non E Thomas, Julien S Baker

**Affiliations:** 1 Health and Exercise Sciences, School of Science and Technology, University of the West of Scotland, Hamilton, UK; 2 School of Human Sciences, Swansea University, Swansea, UK

## Abstract

**Background:**

Cardiovascular disease (CVD) is the main cause of mortality throughout the world. With accumulating evidence suggesting that CVD has its origins in childhood, it is unsurprising that research into obesity prevalence within school aged youth is burgeoning.

Within this study our primary objective will be to examine whether high intensity interval training (HIT) improves the CVD risk profile of secondary school aged adolescents. Our secondary objective will be to identify the prevalence of CVD risk factors and examine factors associated with these in adolescents aged 15 - 18 years.

**Method/Design:**

A South Lanarkshire school of low socioeconomic status (SES) was selected to participate in the study intervention. Participants from secondary 5 (15 - 17 years) and 6 (16 - 18 years) will be recruited for this study. Participants from secondary 6 will be randomly assigned to Group A (HIT) or Group B (moderate-vigorous) and will perform each protocol three times weekly. The secondary 5 participants will act as the control group. Data collection will take place during the Physical Education (PE) lessons and on school premises and will include: anthropometrical variables (height, weight, waist and hip circumferences, skinfold thickness at two sites), physiological responses (blood pressure, aerobic fitness, heart rate (HR) response, vertical jump performance, 10-metre (m) sprint, 50-m sprint and 505-agility test), diet (self-reported seven-day food diary), physical activity (Physical Activity Questionnaire for Adolescents (PAQ-A)) and blood tests (fasting glucose, insulin, total cholesterol (TC), high-density lipoprotein (HDL), high-sensitivity C-reactive protein (hs-CRP), fibrinogen (Fg), interleukin-6 (IL-6), adiponectin (high molecular weight), triglyceride and plasminogen activator inhibitor-1 (PAI-1). An environmental audit of the secondary school and the health related quality of life (HRQOL) of the participants will also be measured. Finally, all exercise sessions will be video recorded and rate of perceived exertion (RPE) and mood states will also be taken after each exercise session.

**Discussion:**

Our study may be able to demonstrate a time efficient means of reducing CVD risk factors in adolescents.

**Trial Registration:**

NCT01027156

## Background

The pandemic rise in overweight and obesity is now very much a global concern. Within the United Kingdom (UK), recent investigations denote worrying figures. In England it is estimated that 25% of adults are obese with an additional 40% overweight whereas in Scotland estimates indicate that 66% of adult males and 60% of adult females are either overweight or obese [1,2]. The findings concerning children and adolescents are equally troubling. Recent estimates from England purport that 14% of 2-15 year olds are obese while 18% are overweight [2]. Within Scotland estimates suggest that for 2-15 year olds, 35% of boys and 30% of girls are either overweight or obese [1]. Given the complexity of factors associated with both overweight and obese status within adults and children it is difficult to predict future trends. Recent findings seem to intimate that obesity levels in England's youth may be beginning to level off [3]. Though this is encouraging, and as the authors [3] contend, it is too early to confirm whether this is an ongoing trend. Others meanwhile suggest that if current trends are to continue unabated in the UK, 60% of men and 50% of women by the year 2050 will be classified as obese [4].

CVD is the main cause of mortality throughout the world with a reported 17.1 million deaths attributed to the disease in 2004, an astonishing 29% of global deaths in that year alone [5]. Though chronic disease may develop over an individual's lifetime, accumulating evidence suggest that CVD has its origins in childhood [6,7]. Findings from the longitudinal Bogulasa Heart Study [8] in the United States (US) found that 60% of 10 year old children who were overweight had at least one biochemical or clinical CVD risk factor. A further 25% had three or more. Considering that childhood overweight is a risk factor for severe obesity throughout life and is associated with morbidity and mortality of several diseases [9], it is unsurprising that research into obesity prevalence within youth is burgeoning. Particularly when recent reports estimate that 10% of all youth globally will become overweight [10] and that 80% of obese adolescents will become obese adults [11].

Numerous risk factors have been linked to the development of CVD. These include poor dietary habits, physical inactivity, obesity and overweight, hypertension and abnormal lipid profiles [6,7]. While these are well established, recent emergent biochemical risk factors have also been identified and include, most notably, elevated levels of hs-CRP, IL-6,; and reduced levels of adiponectin (high molecular weight) [12,13]. However, a number of these risk factors are modifiable.

Nevertheless, the relationship between physical fitness, PA, CVD risk factors, overweight and obesity is poorly understood in youth. Despite gathering evidence [14,15], there is still a need for further research that can strengthen this relationship, particularly as current PA recommendations for youth have been questioned [6]. This will not only help identify those in danger of future CVD but will also provide a means of assessing the effectiveness of future interventions.

### Physical activity and obesity prevention

Understanding the underlying factors and mechanisms whereby individuals become overweight or obese is challenging. There are a wide range of behavioural, genetic, cultural, environmental and biological variables that act both independently and amongst one another which contribute to the development of overweight and obesity in youth [16]. Nonetheless, it is generally accepted that two modifiable behavioural factors have contributed significantly to the aetiology of this current pandemic: dietary habits and PA [17,18]. While we fully acknowledge the key role of dietary intake in the aetiology of overweight and obesity, this study will primarily focus on modifying PA behaviour.

Despite the well established benefits of regular PA on health and well-being, current levels of PA in youth are widely regarded as insufficient to meet current recommendations [19]. Recent findings from the US purport that only 8% of adolescents (12 - 19 years of age) are sufficiently active to meet these recommendations [20]. In Europe, only one third of adolescents (11 - 15 years of age) meet the UK guidelines of 1 hour of moderate to vigorous intensity physical activity (MVPA) most days [21]. Though these findings are perturbing, caution in its interpretation is warranted.

For instance, PA in youth can occur in a variety of situations and contexts - through active commuting, recreation, school and in the household [22]. Given that the above author [22] has questioned the reliability, objectivity and validity of many of the methods used in assessing PA behaviour, current summations are inherently imprecise. Though evidence is gathering through advancements in technology and the use of more objective measures [23,24], much needed research is warranted. Until then, the extent of which youth of different age, gender and cultural climates are meeting current PA recommendations will remain unclear.

### Combating overweight and obesity in school age youth

It is now well established that PA behaviours are influenced by a range of various influences and as such, a multilevel approach targeting these influences are warranted [25]. This switch in focus however is relatively recent and at present, much is still to be learned of the multiple influences on PA behaviour. It has been suggested that in the past, researchers were too reliant on the dominant models and theories of behaviour which emphasized the importance of psychological and social influences [26]. While both of these influences are important, the field of PA research has now advanced by recognizing the importance of factors at multiple levels. This it seems has stemmed from the success of smoking cessation rates in the US from 1965 - 2005. This success has been attributed to a number of multi-level population based health behaviour interventions, all of which have been informed by the principles afforded by ecological models [27].

Ecological models profess that individual, interpersonal, organizational, societal and community factors should be considered when planning and implementing health promotion interventions [28]. It is unsurprising that traditional PA interventions rooted in a cognitive-rational paradigm which focus on changing behaviour at an individual level have demonstrated limited long-term success [29]. Though these interventions often alter behaviour initially, it is suggested that enduring social and environmental factors are more likely to sustain healthy PA behaviours [30]. Thus, interventions that can be directed at the whole community and encompass as large a percentage of the population must be the setting where future multilevel interventions are implemented. Schools afford the ideal setting to practice health-promoting behaviours, and are a critical part of the social environment that shape future behaviours [31]. With this in mind, the potential therefore of PE within schools to affect youth activity levels should not be dismissed.

### Physical activity and cardiovascular disease: the role of physical education

PE is available to most individuals and can provide a safe, structured context for regular PA. Despite this, evidence suggest that children and adolescents spend less that 50% of PE time in moderate intensity activity thus failing to procure any health related benefits [32]. While some have questioned the influence PE can have on overall activity levels [33], some suggest that activity levels would be even lower were youth not exposed to any [34]. What is becoming more evident nonetheless is that the intensity of PE classes are inappropriate to induce health related benefits [32]. The reasons for this are complex [33] but if PE is to be the only exposure to PA for some individuals, this environment has to enhance the health and well-being of those exposed to it. The challenge for practitioners therefore is to ensure that the stimulus provided is appropriate to induce health and well-being in all exposed individuals. Unfortunately, measuring the influence of PE on health and well-being is challenging.

The comparison of PA levels across the PE curriculum is nigh impossible due to confounding variables such as lesson delivery, teaching personnel, environment, equipment, motivation, skill ability, class size and time restrictions [35]. This is in addition to the difficulties associated with accurately measuring the intensity of activity. Recent advancements in technology has seen accelerometers being touted as an objective measure of exercise intensity by some authors [19,36]. However, accelerometers are unable to delineate between different behaviours such as walking or running. Understandably the relationship between school PE and health has been impossible to quantify. With the role of PE continually being marginalized within the educational curriculum, its justification as a contributor to health and fitness in youth is debatable.

Regrettably, the duration, type, and intensity of activity necessary to illicit cardiovascular and psychological health benefits in youth remains equivocal. There is insufficient clinical evidence to confirm that the recommendations proposed for children and adolescents (one hour per day of moderate activity) [37] has any short or long-term health benefits [38]. It is widely accepted that regular PA of this nature is necessary to accrue health benefits and prevent future CVD; nevertheless, the majority of youth fail to meet these current guidelines. Given that lack of time is commonly cited as a key determinant of being physically active regardless of age, gender or ethnicity [39] suggests that current recommendations may be inappropriate for youth.

To our knowledge there has been no empirical study that has directly compared how training strategies might affect the health status in youth. Accordingly, additional research seems warranted to determine whether there is a more time- efficient method of improving the health and fitness of youth. Recent findings suggest that brief intense, interval-based exercise interventions may stimulate cardiovascular adaptations and provide protection against future and current CVD [40-45]. Therefore, we propose a time efficient training regime which can be performed concurrently with the national curriculum and that will afford the measurement of health related outcomes. In particular, this study will aim to identify a time-efficient PA strategy for improving the health profile, CVD pre-cursers, overweight and obesity levels of youth in the UK.

We fully acknowledge that for this intervention to be successful, appreciation of the multiple influences upon behaviour is required. Indeed, a multilevel approach is needed for implementing population based health behaviour interventions that can enhance the health and well-being of individuals [25]. Thus, this pilot intervention will be influenced by the key principles afforded by ecological models. The findings of this study will be used to guide and enhance the effectiveness of future interventions at multiple levels within a school environment.

## Method/Design

### Objectives

This study will aim to implement a 7 week school based HIT intervention that will seek to enhance our understanding of the relationship between exercise intensity and health status in youth.

### Primary objectives

1. To reduce measures of obesity including body mass index (BMI), total skinfold thickness and waist and hip circumferences.

2. To reduce systolic and diastolic blood pressure.

3. To improve aerobic profiles and increase PA levels.

4. To improve blood lipid profiles and lower levels of plasma inflammatory markers.

5. To assess the impact of the HIT intervention on physiological responses.

6. To assess the role of a secondary high school as a setting for promoting healthy eating and PA behaviours.

7. To improve understanding of the multiple levels of influence on behaviour.

### Secondary objectives

1. To determine the associations between CVD risk factors at baseline in 15 - 18 year old youth. Investigate the relationship between specific measures within, and between participants.

### Participants

A South Lanarkshire school of low socio-economic status (SES) was selected to participate in the study. Free school meal eligibility was used to determine SES. This method has previously been validated as an accurate and valid measure of SES in children [46] and as such, allowed the researcher to objectively distinguish between low and high SES groups. Following initial discussions with the school principal and relevant teaching staff, written consent from the principal will be attained. Upon receipt, members of the research team will visit the intended participants from secondary 5 (15 - 17 years) and secondary 6 (16 - 18 years) to discuss the project. Thereafter, information sheets, participant assent forms and parental/guardian consent forms will be distributed. Only the participants who returned both the assent and consent forms will be eligible to participate in the study. Ethics approval was granted by the University of the West of Scotland Ethics committee.

### Data Analysis

A pre- and post-intervention design will be used to compare differences in measured outcomes between participants assigned to the control group and the intervention groups. Initially, baseline data will be used to perform Pearson's product moment correlation coefficients, followed by multiple regression analysis to estimate the significance of selected predictors on CVD risk factors. Statistical significance will be set at *P *= 0.05.

### Intervention

Participants involved in the intervention will be assigned to groups A or B by a third party using computer generated sequences of random numbers. The research team and the participants will be blinded to the randomization process, and assignment to groups will only be disclosed after baseline testing. All participants will be instructed not to change their dietary or lifestyle habits other than prescribed. The secondary 5 participants will act as the control group and will be assigned to group C (*n *= 24). Participants will be encouraged to continue their regular PA patterns.

**Figure 1:**
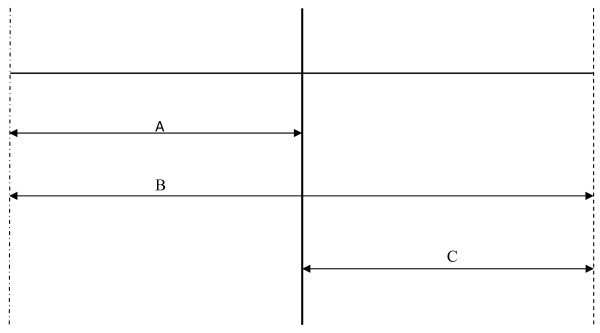
**Course outline showing distance and direction taken by participants, during the 30 s HIT protocol**. A = 10 m, B = 20 m, C = 10 m.

Group A (*n *= 24) will be instructed to complete a 30 seconds (s) maximal effort sprint within a 20 m distance separated by cones (see Figure 1). The start point will be located at the mid-point of the markers. Participants will be instructed to sprint from the midpoint to the first marker, turn, and then sprint 20 m in the opposite direction to the second marker. Thereafter, participants will turn and run again through the midpoint covering a total distance of 40 m. Though this protocol has been used and validated as a measure of anaerobic performance [47], in this study, participants will be instructed to sprint maximally for a period of 30 seconds. Following 30 s rest, the participants will be instructed to repeat this procedure a further 3 times. This equates to 2 minutes (mins) of maximal effort sprinting interspersed with 2 mins recovery. Participants will be requested to perform this protocol 3 times weekly. Training progression will be implemented by increasing the number of repeats from four repetitions during weeks 1 and 2, to five repetitions during weeks 3 and 4, to six repetitions during weeks 5 and 6. Finally, during week 7 participants will still perform six repetitions but interspersed by only 20 s recovery. Prior to the commencement of the training regime, participants will be given a familiarization trial of four low intensity runs under test procedures.

Group B (n = 24) will be instructed to exercise at an intensity of 70% VO_2 _max (moderate) [48], running steadily for a period of 20 mins. The speed of exercise will be determined by the participant's performance in the 20-m shuttle test (MSFT) [49]. During week 4 all participants will be instructed to perform the 20MST to determine any training effect. Upon analysing the participant's performance in week 4, week's 5 - 7 will see the participants instructed to exercise at an intensity that elicits 70% of predicted VO_2_max, running steadily for 20 mins. Finally, RPE using the 15 point scale [50] and mood state will be measured upon the completion of each exercise session.

The intervention will be carried out during the lunch hour or PE lessons, on Monday, Wednesday and Friday, and for a period of 7 weeks. The intervention will be performed in the school sports hall during October through December, 2009, to allow for uninterrupted use of the facilities. Each training day will be supervised by two members of the PE department and at least one researcher. This will ensure that participants continue exercising at the pre-determined intensity. Participants will be instructed to wear appropriate footwear whilst taking part in the intervention. Attendance will be monitored by the researcher on all days when the intervention is scheduled to take place.

### Measurements

All measurements will be performed pre- and post-intervention for both intervention and control groups. In addition, the vertical jump, 10 m sprint, 20MSFT and the 505-agility test will also be taken at the end of week 4 in the intervention group. All measurements will be carried out by experienced individuals during normal PE lessons and on school premises to ensure minimal disruption to the curriculum. Finally, all individuals involved in performing measurements will be CRB checked prior to the commencement of the study.

#### Anthropometrics

Stature will be measured without shoes to the nearest millimetre with the use of a transportable stadiometer (Seca Stadiometer, Seca Ltd, Birmingham, UK). Body mass will be measured in normal PE clothing to the nearest 0.1 kg using a calibrated electronic weighing scales (Seca 880, Digital Scales, Seca Ltd, Birmingham, UK). Body mass index (BMI) will then be calculated by dividing the participants weight by their height squared. Waist and hip circumference will be measured on each participant in an upright position by using anthropometric tape (Rosscraft, UK). Waist circumference will be taken from the narrowest part of the trunk at the end of a gentle expiration. Hip circumference will be taken around the maximum protrusion of the buttocks. Body fat percentage will be estimated from skinfold measurements taken at two sites (triceps and calf) in accordance with standard procedures [51]. A third skinfold measurement will be taken if the first two measurements differ by more than 1.0 mm.

#### Physiological measurements

Systolic and diastolic blood pressure (BP) measurements will be taken using an automated BP monitor (Omron Healthcare UK Ltd, Milton Keynes, UK) after each participant has sat quietly for a period of 10 mins. The cuff will be placed tightly on the upper left arm of the participant with three measurements taken to ensure accuracy. An average of the second and third measures will then be recorded and used for the data analysis [52].

Physical fitness is well established as a marker of health status in youth [6]. Thus, the health related physical fitness components of muscular strength, speed, agility and cardiorespiratory fitness will be measured prior, during and after the intervention.

Heart rate (HR) response will be measured on all participants at the end of each training week through the use of continuous heart rate telemetry (Hosand, TM200, Hosand Technology, Verbania, Italy). The rationale for this will be two fold. Firstly, from this recording the average intensity of each training session will be estimated as a function of a percentage of the theoretical HR peak, in accordance with standard procedures [53]. Secondly, during each weekly session a projector will beam the HR responses onto a wall in the sports hall. Thus all participants will be able to monitor their own HR response during, and after, the exercise session. Upon completion of the exercise session participants will be provided with personalized feedback and will be encouraged to ask questions. Based on previous literature [54,55], it is expected that this personalized feedback will encourage engagement and adherence with the intervention and aims of the study.

Aerobic fitness will be measured through the use of the 20MSFT [49]. The 20MSFT has been validated as a predictor of maximal aerobic power in young people [56] and is familiar with all youth. Participants will be instructed to run between two lines separated by 20 m, while keeping pace with the audio signals emitted from a CD produced by the National Coaching Foundation. The initial speed will be set at 8.5 km/hr which will increase by 0.5 km/hr each minute (1 minute equates to 1 level). All participants will be instructed to continue for as long as possible until they have reached their maximal effort. Otherwise, the test will end when the participant fails to reach the end lines before the audio signal on two consecutive occasions. The CD will be calibrated over a 1 minute duration before its use. Finally, participants will be instructed to complete a standardized warm up and cool down comprising of light jogging and stretching before and after the test and each training session.

The counter movement jump test will be used to measure explosive muscular power. Jumping height will be measured through the use of the Optojump system (Microgate, Bolzano, Italy) after a standardized warm up. Participants will begin in a standing position with their feet shoulder width apart. Thereafter, they will be instructed to perform a counter - movement with their legs prior to jumping. Each participant will be instructed to perform three jumps with a minimum of two mins recovery between each effort. The best of the three jumps (to the nearest cm) will be used as the criterion measure. Participants will be instructed to keep their hands on their hips throughout all jumps, to give maximal effort and land in an extended position. Muscular strength will be measured by the number of completed push ups in 30 s [57].

Sprint speed will be measured over 10 m and 50 m through the use of an electronic sprint timer with photoelectric sensors (Polifemo Radio Light - Microgate, Italy). The 10 m sprint will take place in the school sports hall with the photoelectric sensors being placed at 0 and 10 m intervals. Participants will be instructed to perform each sprint from a standing start. All participants will complete three trials with the fastest time (to the nearest 0.01 s) being used as the criterion measure. Due to area restrictions, the 50 m sprint will take place outdoors on an artificial turf soccer field with the photoelectric sensors being placed at 0 and 50 m intervals. Participants will be instructed to perform each sprint from a standing start. All participants will complete three trials with the fastest time (to the nearest 0.01 s) being used as the criterion measure.

Agility will be measured through the use of the 505 test [58]. Two photoelectric sensors (Polifemo Radio Light - Microgate, Italy) will be placed 5 m from the starting line and 5 m from a designated turning point. Participants will be instructed to sprint maximally to the designated turning point, pivot, and then return as quickly as possible through the photoelectric sensors in accordance with standard procedures [58]. Participants will be instructed to perform three sprints with a minimum of 2 mins recovery between each effort. Times will be measured to the nearest 0.01 s with the fastest value obtained from the three efforts used as the criterion measure.

#### Blood sampling and analysis

Blood samples will be collected between 9:00 am and 11:00 am after an overnight fast. Prior to sampling participants will be instructed to sit quietly for a period of at least 30 mins to control for plasma volume shifts [59]. A team of qualified phlebotomists, experienced in paediatric sampling techniques will take blood samples from the participants. Participants will be provided with breakfast in the school canteen once sampling is complete. The samples will be analyzed for total cholesterol (TC), insulin, high-density lipoprotein (HDL), high-sensitivity C-reactive protein (hs-CRP), glucose, fibrinogen (Fg), interleukin-6 (IL-6), adiponectin (high molecular weight), triglyceride and plasminogen activator inhibitor-1 (PAI-1). Within four hours all blood samples are to be transported to the laboratory where they will be aliquoted and frozen at - 80°C.

#### Questionnaires

All participants will be instructed to complete the physical activity questionnaire for adolescents (PAQ-A). This validated questionnaire has been used in previous research [60] with participants instructed to recall their PA behavior from the previous 7 days. From previous experience we envisage that completion of the questionnaire will take no longer than 30 minutes and thus can be completed during scheduled class time. Sexual maturation status will be determined through the use of a self-reported questionnaire [61]. Participants will be asked to indicate their current stage of development regarding their pubic hair and genital development. Health related quality of life (HRQOL) will be measured through the use of the paediatric quality of life inventory (PedsQL™) for both the participant and their parent/guardian [62]. The PedsQL™ has been shown to be a valid and reliable instrument specifically developed for use with children and adolescents [63]. The brief one page instrument has 23 questions and covers four dimensions: physical functioning, emotional functioning, social functioning and school functioning.

#### Dietary intake

Daily food intake will be monitored through the use of a validated, self-reported food diary [64] and a food frequency questionnaire. Completion of the food diary will require participants to record everything they eat and drink over a specified seven-day period. In order to clarify the foods and drinks that are consumed, the food frequency questionnaire will include 12 semi-quantitative items. Participants will be asked to complete the questionnaire and food diary in their own home after a 45 minute oral training session to help describe intake and estimate portion size. The collated data will be analyzed using nutritional analysis software by Health Options Ltd (Nutri Check, Health Options Ltd, Cirencester, Gloucester, UK). Values for the nutrient amounts in foods will be obtained from McCance and Widdowson's the Composition of Foods [64]. Participants will be encouraged to continue their normal eating and drinking habits throughout the intervention.

#### Environmental audit

The Secondary School Environmental Audit tool [65] will be used to assess the school as a setting for promoting healthy eating and PA. This will include assessment of the physical (what is available), economic (what are the financial factors), policy (what are the rules) and socio-cultural (what are the attitudes, beliefs and perceptions) environments in relation to nutrition, PA and the promotion of healthy body size. The self completing audit tool comes in three parts with the relevant part being sent to the deputy principal, canteen manager and three teachers as recommended [65]. Preventing overweight and obesity requires understanding and changing of the obesogenic (obesity promoting) environment in which individuals frequent. Thus, this audit will be able to identify environmental factors that promote unhealthy weight gain and help inform future interventions.

#### Observational analysis

Observational data will be gathered by video recording all sessions. Direct behavioural observation can provide contextually rich data and is cited as the criterion measure of paediatric PA behaviour [66]. By doing so we will be able to see not only quantitative measurements such as distance covered and the variability of pace within and between the 30 s maximal effort sprints, but also how the exercise protocols affect participants through different communication, responses, behaviours and actions. In addition, critical moments such as 'drop out' could be assessed and evaluated. Finally, with the synchronisation of video with HR, effects of various constraints such as feedback, peer, teacher and researcher influence could be evaluated within, between and across sessions.

#### Feedback to participants

This feedback will include the results of all pre and post measurements; how individual results compare to the desirable level for this age group; and guidance on how unfavourable measures might be improved through lifestyle changes. Feedback will also be given to relevant teaching staff, on group, but not individual, findings. This will provide the school with invaluable information on the general health and well-being of their pupils which can help inform future interventions.

#### Sample size

In order to determine sensitivity to change, sample size will be estimated using the procedures of Park and Schutz [67] for ANOVA designs that incorporate a repeated factor. For a medium effect size in the intervention groups of d = 0.50, power of .80 (suggesting an 80% probability of achieving significance at the *p *= .05 level) this is achieved with a group size of 17 participants. Effect sizes > 0.5 indicate clinically relevant changes [68]. Assuming a drop-out rate of 30%, this would require an initial recruitment of 24 participants per group. The intervention will be carried out during the lunch hour or PE lessons, on Monday, Wednesday and Friday, and for a period of 8 weeks. The interventions will be performed in the school sports hall; led initially by the research team and then by the PE teacher; and during January through May, 2009, to allow for uninterrupted use of the facilities.

## Discussion

It is well established that regular PA has beneficial effects on the health and well-being of youth [37]. Nevertheless, current levels of PA are widely regarded as insufficient to meet current guidelines[19], with lack of time often cited as a contributing factor[43]. Health promotion interventions that aim to modify environmental factors that influence behaviour have now become an integral focus of emerging research being undertaken in the school environment. We fully embrace the concepts afforded through ecological models and have thus developed a comprehensive intervention approach that will target several influences of behaviour. Thus, in this study we aim to provide a comprehensive school based intervention strategy that will aim to determine whether HIT can elicit CVD protection in adolescents.

## Competing interests

The authors declare that they have no competing interests.

## Authors' contributions

JB and NT designed and wrote the original proposal. DB and SO led the overall process. DB led the writing for this manuscript with contributions and critical comment from the other three authors. All authors read and approved the manuscript.

## Pre-publication history

The pre-publication history for this paper can be accessed here:

http://www.biomedcentral.com/1471-2458/10/8/prepub

## References

[B1] Scottish Executive The Scottish Health Survey 2003 Edinburgh: The Scottish Executive

[B2] Department of Health Choosing activity: a physical activity action plan 2005 London: Department of Health

[B3] British Heart Foundation Couch Kids: The Nation's Future... 2009 London: British Heart Foundation

[B4] Information Centre for Health and Social Care Statistics on Obesity, Physical Activity and Diet: England 2008 Information Centre for Health and Social Care

[B5] World Health Organization Preventing chronic diseases a vital investment 2005 Switzerland: World Health Organization

[B6] Andersen LB Harro M Sardinha LB Froberg K Ekelund U Brage S Anderssen SA Physical activity and clustered cardiovascular risk in children: a cross-sectional study (The European Youth Heart Study) Lancet 2006 368 9532 299 304 10.1016/S0140-6736(06)69075-2 16860699

[B7] Eisenmann JC Welk GJ Wickel EE Blair SN Stability of variables associated with the metabolic syndrome from adolescence to adulthood: the Aerobics Center Longitudinal Study American Journal of Human Biology 2004 16 6 690 696 10.1002/ajhb.20079 15495227

[B8] Berenson GS Srinivasan SR Bao W Newman WP Tracy RE Wattigney WA Association between multiple cardiovascular risk factors and atherosclerosis in children and young adults. The Bogalusa Heart Study New England Journal of Medicine 1998 338 23 1650 1656 10.1056/NEJM199806043382302 9614255

[B9] Ferraro K Thorpe R Jr Wilkinson J The life course of severe obesity: does childhood overweight matter? The Journals of Gerentology: Series B: Psychological sciences and social sciences 2003 58 2 S110 S119 10.1093/geronb/58.2.s110 PMC3358723 12646600

[B10] Lobstein T Obesity in children British Medical Journal 2008 337 a669 10.1136/bmj.a669 18719009

[B11] Schonfeld-Warden N Warden CH Pediatric obesity. An overview of etiology and treatment Pediatric Clinics of North America 1997 44 2 339 361 10.1016/S0031-3955(05)70480-6 9130924

[B12] Thomas NE Cooper SM Williams SR Baker JS Davies B Fibrinogen, homocyst(e)ine, and C-reactive protein concentrations relative to sex and socioeconomic status in British young people American Journal of Human Biology 2005 17 6 809 813 10.1002/ajhb.20447 16254908

[B13] Rees A Thomas N Brophy S Knox G Williams R Cross sectional study of childhood obesity and prevalence of risk factors for cardiovascular disease and diabetes in children aged 11-13 BMC public health 2009 9 86 10.1186/1471-2458-9-86 19317914 PMC2667418

[B14] Andersen LB Wedderkopp N Hansen HS Cooper AR Froberg K Biological cardiovascular risk factors cluster in Danish children and adolescents: the European Youth Heart Study Preventive Medicine 2003 37 4 363 367 10.1016/S0091-7435(03)00145-2 14507494

[B15] Thomas NE Baker JS Graham MR Cooper SM Davies B C-reactive protein in schoolchildren and its relation to adiposity, physical activity, aerobic fitness and habitual diet British journal of sports medicine 2008 42 5 357 360 10.1136/bjsm.2007.043604 18178678

[B16] Story M Sallis JF Orleans CT Adolescent obesity: towards evidence-based policy and environmental solutions Journal of Adolescent Health 2009 45 3 Suppl S1 5 10.1016/j.jadohealth.2009.06.022 19699432

[B17] Sallis JF Prochaska JJ Taylor WC A review of correlates of physical activity of children and adolescents Medicine & Science in Sports & Exercise 2000 32 5 963 975 10.1097/00005768-200005000-00014 10795788

[B18] Sacks G Swinburn B Lawrence M Obesity Policy Action framework and analysis grids for a comprehensive policy approach to reducing obesity Obesity Reviews 2009 10 1 76 86 10.1111/j.1467-789X.2008.00524.x 18761640

[B19] McLure SA Summerbell CD Reilly JJ Objectively measured habitual physical activity in a highly obesogenic environment Child Care Health Development 2009 35 3 369 375 10.1111/j.1365-2214.2009.00946.x 19397599

[B20] Troiano RP Berrigan D Dodd KW Masse LC Tilert T McDowell M Physical activity in the United States measured by accelerometer Medicine and Science in Sports and Exercise 2008 40 1 181 188 18091006 10.1249/mss.0b013e31815a51b3

[B21] Armstrong N Welsman JR The physical activity patterns of European youth with reference to methods of assessment Sports medicine (Auckland, NZ) 2006 36 12 1067 1086 10.2165/00007256-200636120-00005 17123328

[B22] Wareham N Physical activity and obesity prevention Obesity Reviews 2007 8 Suppl 1 109 114 10.1111/j.1467-789X.2007.00328.x 17316312

[B23] Riddoch CJ Mattocks C Deere K Saunders J Kirkby J Tilling K Leary SD Blair SN Ness AR Objective measurement of levels and patterns of physical activity Archives of disease in childhood 2007 92 11 963 969 10.1136/adc.2006.112136 17855437 PMC2083612

[B24] Metcalf BS Voss LD Hosking J Jeffery AN Wilkin TJ Physical activity at the government-recommended level and obesity-related health outcomes: a longitudinal study (Early Bird 37) Archives of disease in childhood 2008 93 9 772 777 10.1136/adc.2007.135012 18591181

[B25] Sallis JF Cervero RB Ascher W Henderson KA Kraft MK Kerr J An ecological approach to creating active living communities Annual review of public health 2006 27 297 322 10.1146/annurev.publhealth.27.021405.102100 16533119

[B26] Sallis JF Saelens BE Frank LD Conway TL Slymen DJ Cain KL Chapman JE Kerr J Neighborhood built environment and income: Examining multiple health outcomes Social Science & Medicine 2009 68 7 1285 93 10.1016/j.socscimed.2009.01.017 PMC3500640 19232809

[B27] Sallis JF Owen N Fisher E Glanz K, Rimer BK, Viswanath K Ecological models of Health Behavior Health Behavior and Health Education: Theory, Research and Practice 2008 4 San Francisco: Jossey-Bass 465 485

[B28] Stokols D Social ecology and behavioral medicine: implications for training, practice, and policy Behavioral Medicine 2000 26 3 129 138 10.1080/08964280009595760 11209593

[B29] Resnicow K Page SE Embracing chaos and complexity: a quantum change for public health American journal of public health 2008 98 8 1382 1389 10.2105/AJPH.2007.129460 18556599 PMC2446457

[B30] Owen N Humpel N Leslie E Bauman A Sallis JF Understanding environmental influences on walking: Review and research agenda American Journal of Preventive Medicine 2004 27 1 67 76 10.1016/j.amepre.2004.03.006 15212778

[B31] Parcel GS Simons-Morton B O'Hara NM Baranowski T Wilson B School promotion of healthful diet and physical activity: impact on learning outcomes and self-reported behavior Health Educaction Quarterly 1989 16 2 181 199 10.1177/109019818901600204 2732062

[B32] Fairclough SJ Stratton G Physical activity levels in middle and high school physical education: a review Pediatric Exercise Science 2005 17 3 217 236

[B33] Fox KR Cooper A McKenna J The School and Promotion of Children's Health-Enhancing Physical Activity: Perspectives from the United Kingdom Journal of Teaching in Physical Education 2004 23 4 338 358

[B34] Trost SG School Physical Education in the Post-Report Era: An Analysis From Public Health Journal of Teaching in Physical Education 2004 23 4 318 337

[B35] Fairclough SJ Stratton G Physical Activity, Fitness, and Affective Responses of Normal-Weight and Overweight Adolescents During Physical Education Pediatric exercise science 2006 18 1 53

[B36] Reilly JJ Penpraze V Hislop J Davies G Grant S Paton JY Objective measurement of physical activity and sedentary behaviour: review with new data Archives of disease in childhood 2008 93 7 614 619 10.1136/adc.2007.133272 18305072

[B37] Strong WB Malina RM Blimkie CJ Daniels SR Dishman RK Gutin B Hergenroeder AC Must A Nixon PA Pivarnik JM Evidence based physical activity for school-age youth Journal of Pediatrics 2005 146 6 732 737 10.1016/j.jpeds.2005.01.055 15973308

[B38] Boreham C Riddoch C The physical activity, fitness and health of children Journal of Sports Sciences 2001 19 12 915 929 10.1080/026404101317108426 11820686

[B39] Godin G Desharnais R Valois R Lepage L Jobin J Bradet R Differences in perceived barriers to exercise between high and low intenders: observations among different populations American Journal of Health Promotion 1994 8 4 279 285

[B40] Wisloff U Stoylen A Loennechen JP Bruvold M Rognmo O Haram PM Tjonna AE Helgerud J Slordahl SA Lee SJ Superior cardiovascular effect of aerobic interval training versus moderate continuous training in heart failure patients: a randomized study Circulation 2007 115 24 3086 3094 10.1161/CIRCULATIONAHA.106.675041 17548726

[B41] Tjonna AE Lee SJ Rognmo O Stolen TO Bye A Haram PM Loennechen JP Al-Share QY Skogvoll E Slordahl SA Aerobic interval training versus continuous moderate exercise as a treatment for the metabolic syndrome: a pilot study Circulation 2008 118 4 346 354 10.1161/CIRCULATIONAHA.108.772822 18606913 PMC2777731

[B42] Burgomaster KA Howarth KR Phillips SM Rakobowchuk M Macdonald MJ McGee SL Gibala MJ Similar metabolic adaptations during exercise after low volume sprint interval and traditional endurance training in humans Journal of Physiology 2008 586 1 151 160 10.1113/jphysiol.2007.142109 17991697 PMC2375551

[B43] Gibala MJ Little JP van Essen M Wilkin GP Burgomaster KA Safdar A Raha S Tarnopolsky MA Short-term sprint interval versus traditional endurance training: similar initial adaptations in human skeletal muscle and exercise performance Journal of Physiology 2006 575 Pt 3 901 911 10.1113/jphysiol.2006.112094 16825308 PMC1995688

[B44] Burgomaster KA Hughes SC Heigenhauser GJ Bradwell SN Gibala MJ Six sessions of sprint interval training increases muscle oxidative potential and cycle endurance capacity in humans Journal of Applied Physiology 2005 98 6 1985 1990 10.1152/japplphysiol.01095.2004 15705728

[B45] Coyle EF Very intense exercise-training is extremely potent and time efficient: a reminder Journal of Applied Physiology 2005 98 6 1983 1984 10.1152/japplphysiol.00215.2005 15894535

[B46] Shuttleworth I The relationship between social deprivation, as measured by individual free British Educational Research Journal 1995 21 4 487 10.1080/0141192950210404

[B47] Baker JS Davies B High intensity exercise assessment: relationships between laboratory and field measures of performance Journal of Sports Science and Medicine 2002 5 4 341 347 10.1016/S1440-2440(02)80022-6 12585617

[B48] Tabata I Nishimura K Kouzaki M Hirai Y Ogita F Miyachi M Yamamoto K Effect of moderate-intensity endurance and high-intensity intermittent training on anaerobic capacity and VO2max Medicine & Science in Sports & Exercise 1996 28 10 1327 1330 10.1097/00005768-199610000-00018 8897392

[B49] Leger LA Mercier D Gadoury C Lambert J The multistage 20 metre shuttle run test for aerobic fitness Journal of Sports Sciences 1988 6 2 93 101 3184250 10.1080/02640418808729800

[B50] Borg G Borg's perceived exertion and pain scales 1998 Champaign: Human Kinetics

[B51] Lohman TG Advances in body composition assessment 1992 Champaign, Ill.: Human Kinetics Publishers

[B52] Paradis G Lambert M O'Loughlin J Lavallee C Aubin J Berthiaume P Ledoux M Delvin EE Levy E Hanley JA The Quebec Child and Adolescent Health and Social Survey: design and methods of a cardiovascular risk factor survey for youth The Canadian Journal of Cardiology 2003 19 5 523 531 12717488

[B53] Robergs RA Landwehr R The surprising history of the "HRmax = 220-age" equation Journal of Exercise Physiology Online 2002 5 2 1 10

[B54] Diclemente CC Marinilli AS Singh M Bellino LE The Role of Feedback in the Process of Health Behavior Change American Journal of Health Behavior 2001 25 3 217 217 11322620 10.5993/ajhb.25.3.8

[B55] McManus AM Masters RS Laukkanen RM Yu CC Sit CH Ling FC Using heart-rate feedback to increase physical activity in children Preventive Medicine 2008 47 4 402 408 10.1016/j.ypmed.2008.06.001 18590757

[B56] Boreham CA Paliczka VJ Nichols AK A comparison of the PWC170 and 20-MST tests of aerobic fitness in adolescent schoolchildren Journal of Sports Medicine and Physical Fitness 1990 30 1 19 23 2366530

[B57] Behm DG Faigenbaum AD Falk B Klentrou P Canadian Society for Exercise Physiology position paper: resistance training in children and adolescents Applied Physiology, Nutrition & Metabolism 2008 33 3 547 561 10.1139/H08-020 18461111

[B58] Draper JA Lancaster MG The 505 test: a test for agility in the horizontal plane Australian Journal of Science & Medicine in Sport 1985 17 1 15 18

[B59] Pronk NP Short term effects of exercise on plasma lipids and lipoproteins in humans Sports medicine (Auckland, NZ) 1993 16 6 431 448 10.2165/00007256-199316060-00006 8303142

[B60] Janz KF Lutuchy EM Wenthe P Levy SM Measuring activity in children and adolescents using self-report: PAQ-C and PAQ-A Medicine & Science in Sports & Exercise 2008 40 4 767 772 10.1249/MSS.0b013e3181620ed1 18317366

[B61] Tanner J Growth at adolescence 1962 Oxford: Blackwell

[B62] Varni JW Burwinkle TM Seid M The PedsQL™ 4.0 as a School Population Health Measure: Feasibility, Reliability, and Validity Quality of Life Research 2006 15 2 203 215 10.1007/s11136-005-1388-z 16468077

[B63] Varni JW Seid M Kurtin PS PedsQL 4.0: reliability and validity of the Pediatric Quality of Life Inventory version 4.0 generic core scales in healthy and patient populations Medical Care 2001 39 8 800 812 10.1097/00005650-200108000-00006 11468499

[B64] Food Standards Agency McCance and Widdowson's the Composition of Foods 2002 6 Cambridge UK: Royal Society of Chemistry

[B65] Swinburn B Egger G Raza F Dissecting obesogenic environments: the development and application of a framework for identifying and prioritizing environmental interventions for obesity Preventive Medicine 1999 29 6 Pt 1 563 570 10.1006/pmed.1999.0585 10600438

[B66] Sirard JR Pate RR Physical activity assessment in children and adolescents Sports medicine (Auckland, NZ) 2001 31 6 439 454 10.2165/00007256-200131060-00004 11394563

[B67] Park I Schutz RW An introduction to latent growth models: analysis of repeated measures physical performance data Research Quarterly for Exercise & Sport 2005 76 2 176 192 10.1080/02701367.2005.10599279 16128485

[B68] Cohen J A power primer Psychological bulletin 1992 112 1 155 159 10.1037/0033-2909.112.1.155 19565683

